# Assessing the risk of prenatal depressive symptoms in Chinese women: an integrated evaluation of serum metabolome, multivitamin supplement intake, and clinical blood indicators

**DOI:** 10.3389/fpsyt.2023.1234461

**Published:** 2024-01-11

**Authors:** Rongrong Yang, Zhenguo Lin, Yanhua Cai, Nan Chen, Ying Zhou, Jie Zhang, Guolin Hong

**Affiliations:** ^1^State Key Laboratory of Vaccines for Infectious Diseases, Xiang An Biomedicine Laboratory, State Key Laboratory of Molecular Vaccinology and Molecular Diagnostics, National Innovation Platform for Industry-Education Integration in Vaccine Research, School of Public Health, Xiamen University, Xiamen, China; ^2^Department of Clinical Medicine, Xiamen Medical College, Xiamen, China; ^3^Department of Obstetrics and Gynecology, Clinical Medical Research Center for Obstetrics and Gynecology Diseases, The First Affiliated Hospital of Xiamen University, Xiamen, China; ^4^Department of Laboratory Medicine, Xiamen Key Laboratory of Genetic Testing, The First Affiliated Hospital of Xiamen University, School of Public Health, Xiamen University, Xiamen, China

**Keywords:** multimodal data, serum metabolomics, Edinburgh postnatal depression scale, biomarkers, nomogram

## Abstract

**Background:**

Prenatal depressive symptoms (PDS) is a serious public health problem. This study aimed to develop an integrated panel and nomogram to assess at-risk populations by examining the association of PDS with the serum metabolome, multivitamin supplement intake, and clinical blood indicators.

**Methods:**

This study comprised 221 pregnant women, categorized into PDS and non-PDS groups based on the Edinburgh postnatal depression scale. The participants were divided into training and test sets according to their enrollment time. We conducted logistic regression analysis to identify risk factors, and employed liquid chromatography/high resolution mass spectrometry-based serum metabolome analysis to identify metabolic biomarkers. Multiple factor analysis was used to combine risk factors, clinical blood indicators and key metabolites, and then a nomogram was developed to estimate the probability of PDS.

**Results:**

We identified 36 important differential serum metabolites as PDS biomarkers, mainly involved in amino acid metabolism and lipid metabolism. Multivitamin intake works as a protective factor for PDS. The nomogram model, including multivitamin intake, HDL-C and three key metabolites (histidine, estrone and valylasparagine), exhibited an AUC of 0.855 in the training set and 0.774 in the test set, and the calibration curves showed good agreement, indicating that the model had good stability.

**Conclusion:**

Our approach integrates multiple models to identify metabolic biomarkers for PDS, ensuring their robustness. Furthermore, the inclusion of dietary factors and clinical blood indicators allows for a comprehensive characterization of each participant. The analysis culminated in an intuitive nomogram based on multimodal data, displaying potential performance in initial PDS risk assessment.

## Introduction

Prenatal depressive symptoms (PDS) are mood disorders primarily characterized by anhedonia and persistent low mood (1–4). The prevalence of PDS has increased rapidly in the last decade, reaching up to 19%–25% in low-income countries and 7%–15% in high-income countries (5). Importantly, PDS leads to many adverse pregnancy outcomes, such as low birth weight, preterm intrauterine growth restriction (6). Additionally, it also serves as a significant predictor of postpartum depression (7). Numerous studies have reported that PDS is associated with many factors, such as poor social support, history of depression, unplanned pregnancy, exposure to violence, passive smoking, and multivitamin supplements (8–10). However, early diagnosis and treatment remain major challenges. Notably, traditional diagnostic methods, such as scales and structured clinical interviews, are susceptible to reporting bias of symptom severity (11). Up to now, there is no reliable molecular marker for prenatal depression. Therefore, there is an urgent need to identify potential measurable biological biomarkers for personalized PDS identification (12–14) and combine multimodal data for more accurate risk assessment that comprehensively reflects human characteristics (15).

Metabolites, endpoint products of the interactions between gene regulation, protein function, and cellular microenvironment, can accurately reflect the state of biological system. Hence, they are widely used as biomarkers for a variety of diseases (16). Measuring the metabolome holds great promise for the identification of potential biomarkers and related pathways of PDS (17). Metabolomic investigations have revealed that metabolic dysregulation, especially in amino acid metabolism, hormone metabolism, and lipid metabolism, plays a pivotal role in the pathophysiology of PDS (18).

Amino acid metabolism is tightly associated with the PDS progression. Significant features of PDS include abnormal levels of glutamate, phenylalanine, tyrosine, and tryptophan, along with the dysfunction of tryptophan and kynurenine pathways (12, 19–22). Some amino acids and their precursors act as the neurotransmitters in the regulation of depressive symptoms. For example, glutamate, an important excitatory neurotransmitter, is usually observed at low levels in the prefrontal cortex of women with late pregnancy or postpartum depression (19). It can be restored in the dorsolateral prefrontal cortex after progesterone treatment (23). Hormonal disruption during perinatal period may alter glutamate signaling. In addition, catecholamine neurotransmitters, including dopamine, epinephrine and norepinephrine, have a strong association with depressive symptoms (24). Phenylalanine is an important precursor for the synthesis of catecholamines and tyrosine. Tyrosine metabolism, catalyzed by tyrosine hydroxylase to produce dopamine, directly regulates numerous physiological functions of the central nervous system (20). Moreover, tryptophan, as the sole precursor of serotonin (5-hydroxytryptophan, 5-HT), is an essential amino acid of dietary origin. It plays a vital role in dietary intake, and its association with PDS prevalence is well-documented (21). A small fraction of tryptophan is synthesized into 5-HT, while the majority is catabolized via the kynurenine pathway, producing several metabolites with excitatory neurotransmission (22).

Beyond amino acids and their derivatives, lipids’ regulatory role on the central nervous system has gained significant attention. Lower plasma phosphatidylcholine (PC) biosynthesis or lower dietary intake is associated with depressive symptoms and preterm birth in African American pregnant women (25). Polyunsaturated fatty acids play an important role in maternal mental health and offspring neurodevelopment. Specifically, decreased levels of ω-3 docosapentaenoic acid (DPA), eicosapentaenoic acid (EPA), docosahexaenoic acid (DHA), and ω-6 arachidonic acid (AA) were found in the placenta of women with PDS and were significantly associated with poorer social-emotional outcomes in infants (10).

Since these metabolites are mostly derived from food, multivitamin supplementation is suggested to alleviate depressive symptoms. Folic acid, an important nutrient during pregnancy, has been observed in higher plasma levels in healthy pregnant women compared with women with antenatal depression in the growing up in Singapore toward healthy outcomes (GUSTO) cohort (26). In addition, vitamin and mineral formulations have been shown to favorably modulate brain function, potentially countering the development of postpartum depression (27). Therefore, given the high recurrence rate of depression and the low medication adherence rate, nutrition-related factors have great potential for preventive or therapeutic agents for depression.

Depressive symptoms are inherently heterogeneous, making PDS screening susceptible to various influences. Traditional scales and structured clinical interviews may not accurately reflect the pathophysiological processes underlying clinical symptoms. Previous metabolome studies have provided many meaningful potential biomarkers, but the results are not consistent (6, 28, 29). This discrepancy might be attributed to differences in study design, study populations and ethnicity. Predicting PDS risk solely based on a single factor is challenging. Therefore, comprehensive studies that incorporate multiple metabolome biomarkers, clinical data, and multivitamin supplementation factors are essential to construct accurate PDS prediction models.

In this study, we aim to screen serum metabolic biomarkers using liquid chromatography/high resolution mass spectrometry-based metabolome approach, and evaluate the predictive value of metabolic biomarkers, clinical, and lifestyle factors for PDS, finally construct a robust risk prediction model for PDS.

## Materials and methods

### Study population

**Figure 1:**
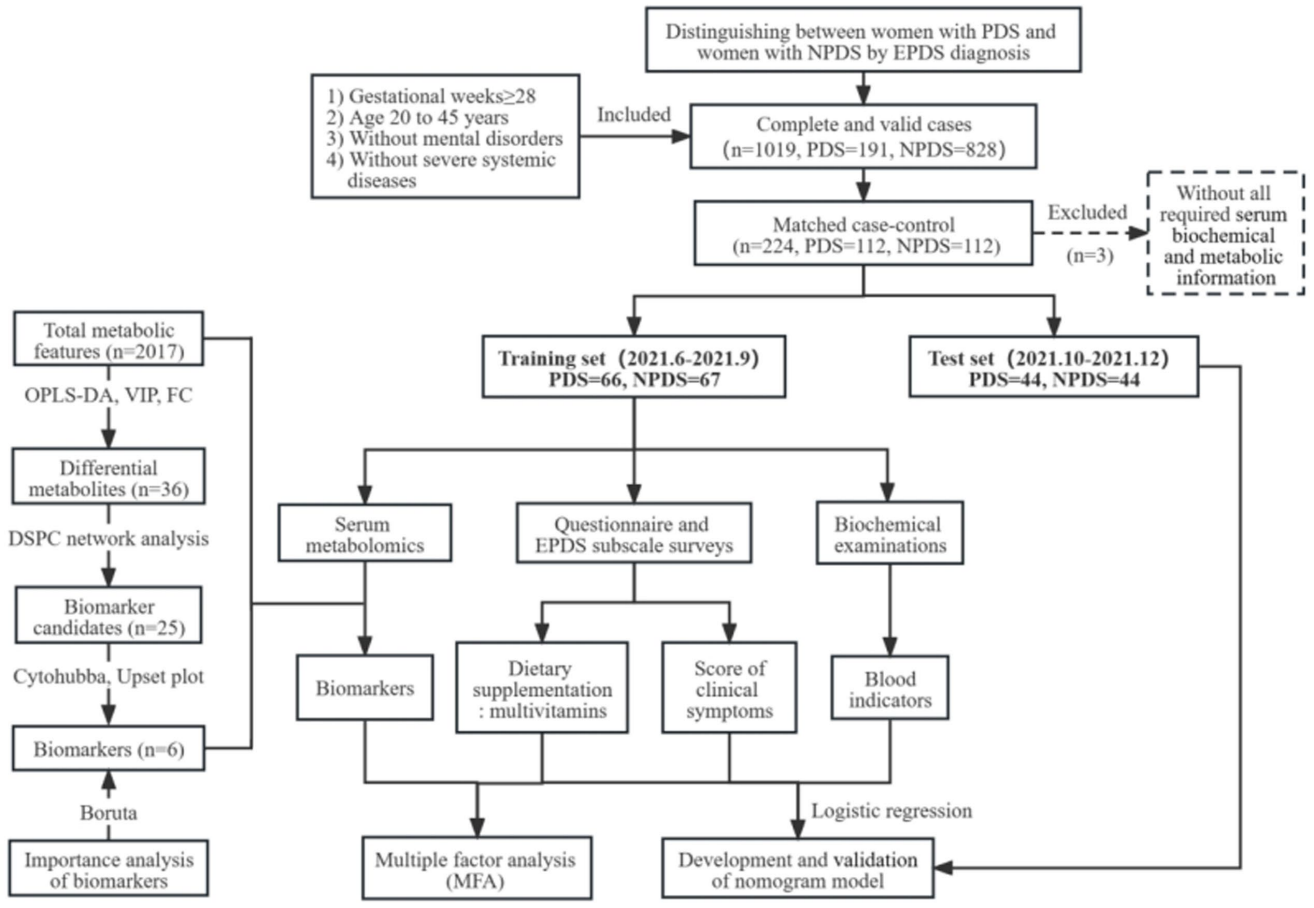
Analytical workflow of this study.

This study was approved by the Medical Ethics Committee of the First Affiliated Hospital of Xiamen University (Approval ID: 2021-Research No. 050). Informed consent was obtained from all participants at the time of recruitment. The workflow of the study design is shown in Figure 1. The study population consisted of pregnant women who received maternity services at the hospital between June 2021 and December 2021. The inclusion criteria were as follows: (1) late pregnancy and gestational age ≥28 weeks; (2) between the ages of 20 and 45 years; (3) planning to give birth in our hospital; (4) without a history of mental or cognitive disorders before pregnancy; (5) without severe systemic diseases such as brain, liver, or kidney diseases and hematopoietic system disease. Women who had a miscarriage or gave birth temporarily elsewhere were excluded before statistical analysis.

All the participants were asked to complete the Edinburgh postnatal depression scale and a face-to-face interview. The scale is a 10-item self-report screening questionnaire for perinatal depression (30), and its Chinese version has demonstrated good reliability and validity (sensitivity, 80.0%; specificity, 83.03%) (31). Each question has four response options ranging from 0–3, with a total score of 30. Subjects were considered to have PDS if the EPDS score was ≥10, and a score of <10 was considered to be non-pregnant depressive symptoms (NPDS). The three subscales of EPDS are anheondonia, anxiety and depressive mood (32). The interview gathered information on demographics, health history, lifestyle, and dietary supplement factors. Demographics included age, pre-pregnancy BMI, pregnancy BMI, gestational week, and parity at birth. The factors were selected according to the previous literature (33–36) on the prediction of the risk of PDS. The lifestyle factors included passive smoking, alcohol consumption, and exposure to chemical substances. Dietary supplements included multivitamins, DHA, iron, zinc, calcium, deep-sea fish, and vegetable oil.

### Clinical blood indicators

Fasting venous blood was collected and centrifuged at 3,000 rpm for 15 min. We measured a number of blood indicators using routine blood biochemistry tests, including alanine aminotransferase (ALT), aspartate aminotransferase (AST), glutamyl transferase (GGT), magnesium glucose (GLU), urea (UREA), creatinine (CRE), total cholesterol (TC), triglycerides (TG), high-density lipoprotein (HDL-C), low-density lipoprotein (LDL-C), glomerular filtration rate (GFR), hemoglobin (HGB), mean red blood cell volume (MCV), prothrombin time (PT), prothrombin time—INR value, activated partial thromboplastin time (APTT), thromboplastin time (TT), fibrinogen (FIB), D-dimer (D-D).

### Non-targeted serum metabolome analysis

Serum was transferred to sterile tubes (2.0 mL) and checked for hemolysis, then non-hemolytic samples were stored at −80°C until metabolomics analysis. Serum proteins were removed by adding 4 volumes of methanol. After centrifugation at 15,000 g and 4°C for 15 min, the supernatant was collected and dried using a Savant Speedvac concentrator. The dried extract was reconstituted in 100 μL of 50% methanol. Five μL of each sample was pooled to make a quality control (QC) sample to evaluate the reproducibility and the stability of the metabolome analysis. A blank sample (50% methanol) was used to evaluate potential background interference.

Serum metabolome acquisition was performed by using high-performance liquid chromatography (HPLC) coupled to high-resolution mass spectrometry (HRMS) (Q-Exactive Orbitrap; Thermo Fisher Scientific, United States). Chromatographic separation was performed using a Waters ACQUITY HSS T3 column (1.8 μm, 100 mm × 2.1 mm i.d.). The temperature of the column and autosampler was set at 50°C and 10°C, respectively. Mobile phase A was Milli-Q water (0.1% formic acid) and mobile phase B was MeOH (0.1% formic acid). The gradient elution program was 0.1% B (0 min), 20% B (2 min), 80% B (15 min), 99.9% B (20 min) and 99.9% B (22 min), at a flow rate of 0.3 mL/min. MS/MS information was acquired under the fragmentation energies of 25, 35 and 45 eV. Metabolomics raw spectra data were subjected to peak extraction, peak alignment and identification using Compound Discoverer 3.1 software (Thermo Fisher Scientific, United States). The output metabolic features was normalized by summing the metabolic data after LOESS correction based on QC samples using the statTarget R package. The metabolites with QC intensity RSD <20% were accepted for metabolome analysis.

### Biomarker screening, nomogram construction and validation

After completion of questionnaire analysis and experimental testing, the participants were divided into training and test sets according to the time of enrollment.

For the training set, multiple models were combined to screen biomarkers. First, differential metabolic features between the PDS and NPDS groups were identified using the orthogonal partial least squares-discriminant analysis (OPLS-DA) model and unified manifold approximation and projection (UMAP). We selected metabolites with variable importance in projection (VIP) values greater than 1 and fold change (FC) greater than 1.2 as differential metabolites. From these differential metabolites, we further refined biomarker candidates using a debiased sparse partial correlation (DSPC) network analysis, which can discover inter-metabolite correlations and identify functionally relevant metabolites. We further explored important metabolites responsible for abnormal regulation of metabolic pathways in PDS using cytohubba (plugins for Cytoscape). Cytohubba provides 12 topological methods such as MCC, DMNC, MNC, degree, EPC, bottleneck, eccentricity, closeness, radiality, betweenness, stress and clustering coefficient. Perturbation maps formed by the intersection of the 12 sets display the final key metabolites as biomarkers. The reliability was validated using Boruta algorithm with 5,000 trees and 500 iterations, which can filter out all significant sets of features that correlate with dependent variable. In addition, we further investigated the relationships between biomarkers, multivitamin supplement factors and subscale symptom scores using multiple factor analysis (MFA). Finally, we constructed a nomogram based on logistic regression, and evaluated its accuracy using the concordance index (C-index), area under curve (AUC), calibration curve, and clinical decision curve (DCA). The model was validated in the test set.

Principal component analysis (PCA) analysis of quality control (QC) clustering were performed using SIMCA14.0. Metabolic enrichment pathways and DSPC network analysis were performed with MetaboAnalyst 5.0.^1^ Cytohubba, based on Cytoscape 3.9.1, was used for network analysis. Other analysis was performed using R 3.6.3. *p*-values less than 0.05 were considered statistically significant.

## Results

### Participant characteristics, lifestyle and multivitamin supplement factors

A total of 221 participants were enrolled in the study, consisting of 110 pregnant women with PDS and 111 with NPDS. Table 1 summarizes the baseline characteristics of the study population. Between the PDS and NPDS groups, there were differences in six blood clinical indicators: TC, TG, HDL-C, LDL-C, GLU and GFR, while no significant difference was observed for age, gestational week, BMI, birth parity, or other indicators.

**Table 1:** Baseline characteristics of the study population.

Characteristics	NPDS (*n* = 111)	PDS (*n* = 110)	*p*
Age^a^	31.83 ± 4.36	30.76 ± 4.52	0.560
Prepregnancy BMI^a^	20.95 ± 3.72	21.03 ± 2.97	0.486
Pregnancy BMI^a^	25.57 ± 3.04	25.87 ± 3.72	0.508
Gestational week^a^	34.72 ± 2.62	34.36 ± 2.46	0.297
Birth parity^b^ *n* (%)			0.943
Yes (firstborn)	59 (53.15)	59 (53.64)	
No	52 (46.85)	51 (46.36)	
Mg^a^	0.78 ± 0.16	0.79 ± 0.09	0.472
CRE^a^	48.87 ± 9.31	47.70 ± 9.59	0.358
TC^a^	7.41 ± 1.59	6.36 ± 1.46	<0.001
TG^a^	5.00 ± 2.80	3.70 ± 1.94	<0.001
HDL-C^a^	1.68 ± 0.60	1.95 ± 0.52	<0.001
LDL-C^a^	4.02 ± 1.20	3.20 ± 1.09	<0.001
GGT^a^	12.57 ± 7.96	13.61 ± 14.10	0.501
GFR^a^	123.78 ± 12.43	123.05 ± 13.16	0.011
INR^a^	0.89 ± 0.06	0.90 ± 0.08	0.213
D-D^a^	9.78 ± 19.92	6.87 ± 17.00	0.243
PT^a^	10.03 ± 0.99	11.12 ± 1.07	0.917
ALT^a^	12.62 ± 6.82	12.23 ± 7.83	0.691
TT^a^	15.53 ± 1.50	15.27 ± 1.75	0.232
AST^a^	17.77 ± 4.74	17.70 ± 5.53	0.917
GLU^a^	5.20 ± 1.28	4.64 ± 1.26	0.001
UREA^a^	3.49 ± 0.96	3.55 ± 1.00	0.614
MCV^a^	91.59 ± 8.43	91.66 ± 6.34	0.950
FIB^a^	4.45 ± 0.85	4.40 ± 0.81	0.706
HGB^a^	123.78 ± 12.43	123.05 ± 13.16	0.669
APTT^a^	26.82 ± 4.30	26.15 ± 3.95	0.229
EPDS score^a^	5.50 ± 2.27	11.65 ± 1.67	<0.001
Anhedonia^a^	1.80 ± 1.22	2.90 ± 0.91	<0.001
Anxiety^a^	2.62 ± 1.34	5.65 ± 1.38	<0.001
Depressive mood^a^	1.08 ± 1.07	3.09 ± 1.12	<0.001

Data presented as mean ± SD or *n* (%).

a Analyzed by the student *t*-test. The results of the corrected *t*-test are presented when the variance is not homogeneous.

b Analyzed by the chi-square test.

Supplementary Table S1 provides baseline information for both the training and test sets. For the training set, we conducted both single-factor logistic regression and multi-factor logistic regression analyses, encompassing all baseline information, lifestyle factors, and dietary supplement factors. In our analysis, we identified multivitamin supplementation as a statistically significant protective factor for PDS [odds ratio (OR) = 0.326, 95% confidence interval (CI): 0.149–0.713]. Supplementary Table S2 shows the coefficients associated with these variables.

### Metabolome biomarker analysis

**Figure 2:**
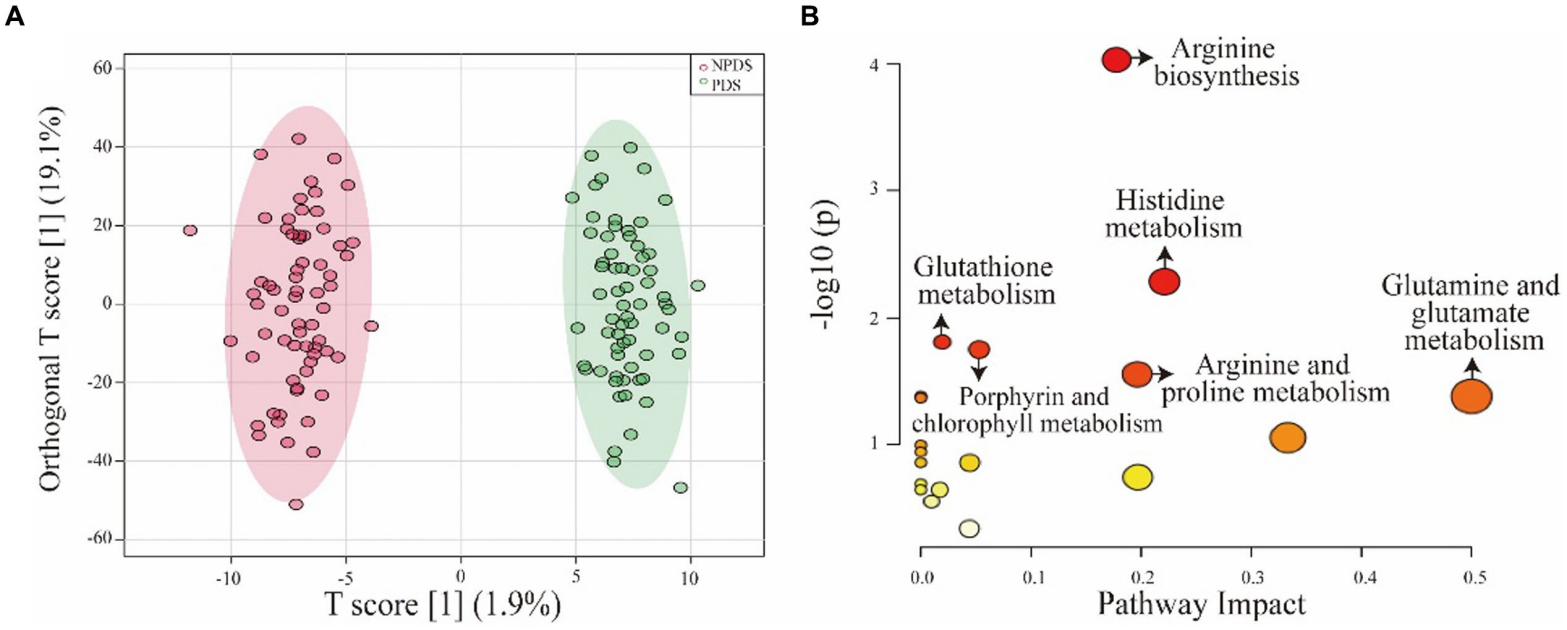
OPLS-DA model and pathway analysis. **(A)** OPLS-DA model showing the significant differences between the two groups. **(B)** Overview of pathway analysis, highlighting the significant metabolic pathways. Significance was determined as *p* < 0.05 and pathway impact >0. The size of the nodes represents the influence factor of a topological analysis, while the color of the nodes indicates the *p*-value of the enrichment analysis.

A total of 2017 metabolic features were extracted from the serum samples. The QC samples were tightly clustered in the PCA plot (Supplementary Figure S1), indicating the robustness of the HPLC-HRMS system. UMAP plot and OPLS-DA model (R2X = 0.44, R2Y = 0.972, Q2 = 0.512) show distinct separation between the PDS and NPDS groups (Supplementary Figure S2; Figure 2A). The permutation test confirmed the robustness of the OPLS-DA model (*p*-values for both Q2 and R2Y were <0.001). The volcano plot visualization (Supplementary Figure S2) of all metabolic features highlighted 36 differential metabolites, which were mainly derived from alkaloids, ketones, organonitrogen compounds, phenylpropanoids, steroid conjugates, and amino acids based on the main chemical structure classification. These differential metabolites were further screened by VIP and FC values (Table 2). KEGG analysis showed 19 enriched pathways (Supplementary Figure S2), including histidine metabolism, porphyrin and chlorophyll metabolism, arginine and proline metabolism, glutamine and glutamate metabolism, glutathione metabolism, arginine biosynthesis (Figure 2B).

**Table 2:** Differential serum metabolites between PDS and NPDS.

**Metabolites**	**HMDB ID**	**VIP**	***p***	**FC** ^**a**^	**FDR**
Beta-tyrosine	HMDB0003831	1.14	1.28E-02	0.83	1.78E-02
Alanylleucine	HMDB0028691	1.19	3.50E-02	1.26	3.82E-02
Glycylvaline	HMDB0028854	1.37	4.36E-03	0.68	8.72E-03
Glutamic acid	HMDB0000148	1.26	1.96E-02	0.83	2.41E-02
Histidine	HMDB0000177	1.50	3.14E-04	0.67	1.73E-03
Ornithine	HMDB0000214	1.77	4.15E-04	0.82	1.73E-03
N(alpha)-benzyloxycarbonyl-L-leucine	NA	1.64	1.62E-03	0.82	4.85E-03
N2-acetylornithine	HMDB0003357	1.62	1.39E-03	0.62	4.53E-03
N-methyl-L-proline	HMDB0094696	1.86	3.56E-04	0.83	1.73E-03
Proline betaine	HMDB0004827	1.46	2.25E-03	2.04	6.22E-03
Alpha-linolenic acid	HMDB0001388	1.15	1.72E-02	0.82	2.21E-02
3-oxotetradecanoic acid	HMDB0010730	1.31	9.48E-03	0.79	1.48E-02
CMPF	HMDB0061112	1.08	2.01E-02	0.73	2.41E-02
Erucamide	HMDB0244507	1.54	2.58E-04	1.27	1.73E-03
Oleamide	HMDB0002117	1.39	2.95E-03	1.29	7.09E-03
17-hydroxypregnenolone sulfate	HMDB0000416	1.50	1.37E-02	0.80	1.83E-02
16α-hydroxy DHEA 3-sulfate	HMDB0062544	1.60	3.78E-03	0.75	8.00E-03
Corchoroside A	HMDB0033846	1.51	3.25E-03	0.77	7.31E-03
Cortolone-3-glucuronide	HMDB0010320	1.15	1.08E-02	0.80	1.55E-02
Estrone	HMDB0000145	1.57	1.10E-03	0.78	3.94E-03
Taurochenodesoxycholic acid	HMDB0000951	1.05	4.82E-02	0.72	4.82E-02
9-*cis*-retinoic acid	HMDB0002369	1.38	2.96E-03	0.77	7.09E-03
LysoPC(16:0/0:0)	HMDB0010382	1.31	4.57E-02	0.80	4.70E-02
Valylasparagine	HMDB0029122	1.26	2.11E-02	0.76	2.45E-02
Trigonelline	HMDB0000875	1.28	6.09E-03	0.81	1.04E-02
12-hydroxydodecanoic acid	HMDB0002059	1.35	6.35E-03	0.79	1.04E-02
Bilirubin	HMDB0000054	1.31	2.37E-02	0.74	2.67E-02
Allantoin	HMDB0000462	1.75	7.00E-05	0.76	8.40E-04
Anandamide (AEA)	HMDB0004080	1.05	4.31E-02	1.22	4.56E-02
Sphingosine	HMDB0000252	1.15	1.01E-02	0.80	1.52E-02
5-decanoyl-2-nonylpyridine	HMDB0035516	1.51	4.33E-04	1.26	1.73E-03
5-O-methylembelin	HMDB0040867	2.54	4.15E-06	0.67	7.47E-05
3,4-dihydroxyhydrocinnamic acid	HMDB0000423	1.74	3.47E-04	0.83	1.73E-03
Caffeic acid 3-O-sulfate	HMDB0041706	2.30	1.03E-07	0.54	3.70E-06
Stachydrine	NA	1.49	5.57E-03	1.90	1.00E-02
Testosterone ketolaurate	NA	1.08	5.22E-03	1.23	9.90E-03

^a^The FC (fold change) was calculated as the ratio between the mean metabolite abundance in PDS relative to NPDS (FC = PDS/NPDS).

**Figure 3:**
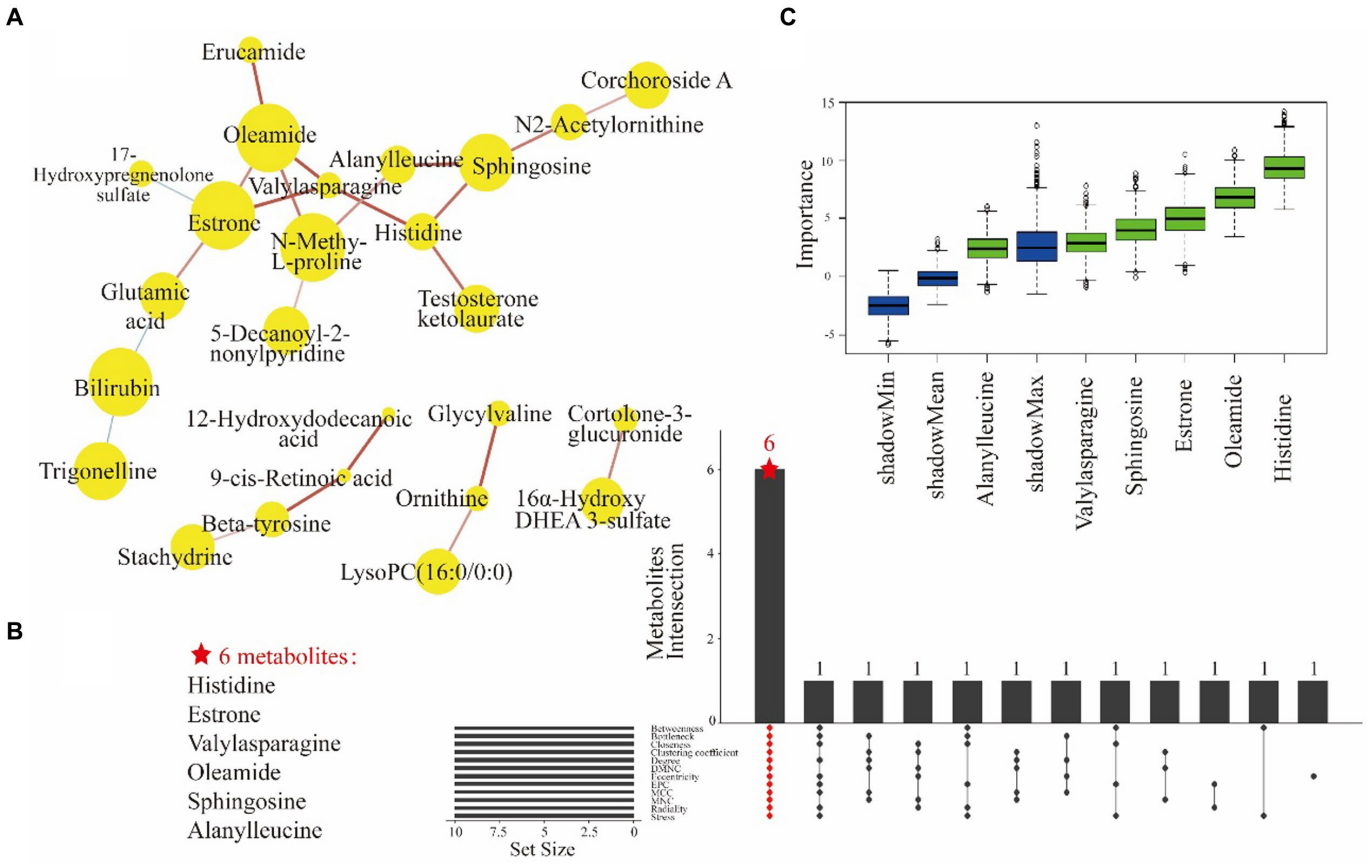
The DSPC network analysis of 36 differential metabolites showed correlations for 25 filtered metabolites **(A)**. Yellow nodes represent metabolites and node size indicates the direction of the change. The lines indicate the partial correlation coefficients of the two connected metabolites after controlling for all other metabolite conditions. Red line and blue line indicate a positive correlation and a negative correlation between two metabolites, respectively. The width of the line indicates the strength of the partial correlation coefficients. The UpSet plot shows the results of 25 metabolites in 12 ranked methods taking intersections to obtain a total of 6 key metabolites **(B)**. The ranked methods are betweenness, bottleneck, closeness, clustering coefficient degree, DMNC, eccentricity, EPC, MCC, MNC, radiality and stress. *Z*-score of metabolite dataset properties under Boruta model **(C)**. *Z*-score as an indirect measure of importance, the blue boxes indicate the minimum, average and maximum *Z*-scores of the shaded attributes. The red boxes (not existing actually) and the green boxes indicate *Z*-score of the rejected and confirmed attributes, respectively.

Moreover, a DSPC network analysis (Figure 3A) was performed for differential metabolites. DSPC allows the intuitive discovery of connectivity among a large number of metabolites (37). The yellow nodes in the graphical model represent metabolites, while the lines indicate the associations between them. Certain amino acids, such as histidine, valylasparagine and alanylleucine, were observed to be clustered together. The centrality of a metabolite within the metabolic network is indicated by its proximity to the center and reflects its strength of correlation with other metabolites and significance. In this study, key metabolites such as histidine, estrone, valylasparagine, alanylleucine, sphingosine, N-methyl-L-proline, and oleamide played central roles as they were involved in multiple metabolic reactions. The DSPC network diagram identified 25 biomarker candidates.

The biomarker candidates were subsequently subjected to centrality calculation using 12 topological methods. The UpSet plots shows that the top 10 metabolites identified by 12 ranking methods had an overlap containing 6 key metabolites: histidine, estrone, valylasparagine, oleamide, sphingosine, and alanylleucine (Figure 3B). These biomarkers were important for the dysregulation of metabolic pathways in PDS. Furthermore, the robustness of these key metabolites in distinguishing PDS was validated through importance analysis of the independent variables using the Boruta model (Figure 3C). Importantly, the significance of these key metabolites was consistently supported by the results from multiple models. Finally, these metabolites were selected to develop a biomarker panel.

### Association of the biomarkers and dietary factors with subscale scores

As shown in Supplementary Figure S3A, the MFA model identified two dimensions that jointly explained 36.44% of the total variation between the PDS and NPDS groups. The symptom scores exerted the greatest influence on the first dimension, while the key metabolites primarily contributed to the second dimension. Supplementary Figure S3B shows a clear separation between the participants taking multivitamins and those not taking multivitamins. Each variable’s quantification is visually represented as a vector extending from the origin to the triangular shape. Supplementary Figure S3D demonstrates that three depressive symptoms (i.e., disorientation, anxiety, and depressed mood) were positively associated with alanylleucine and oleamide, while negatively associated with histidine, estrone, sphingosine and valylasparagine. Supplementary Figures S3C,D show that three depressive symptoms had an obtuse angle relationship with multivitamin intake, indicating a negative correlation. This means that those who had higher intake of multivitamins were likely to have lesser symptoms of depression.

### Construction of a nomogram prediction model

**Figure 4:**
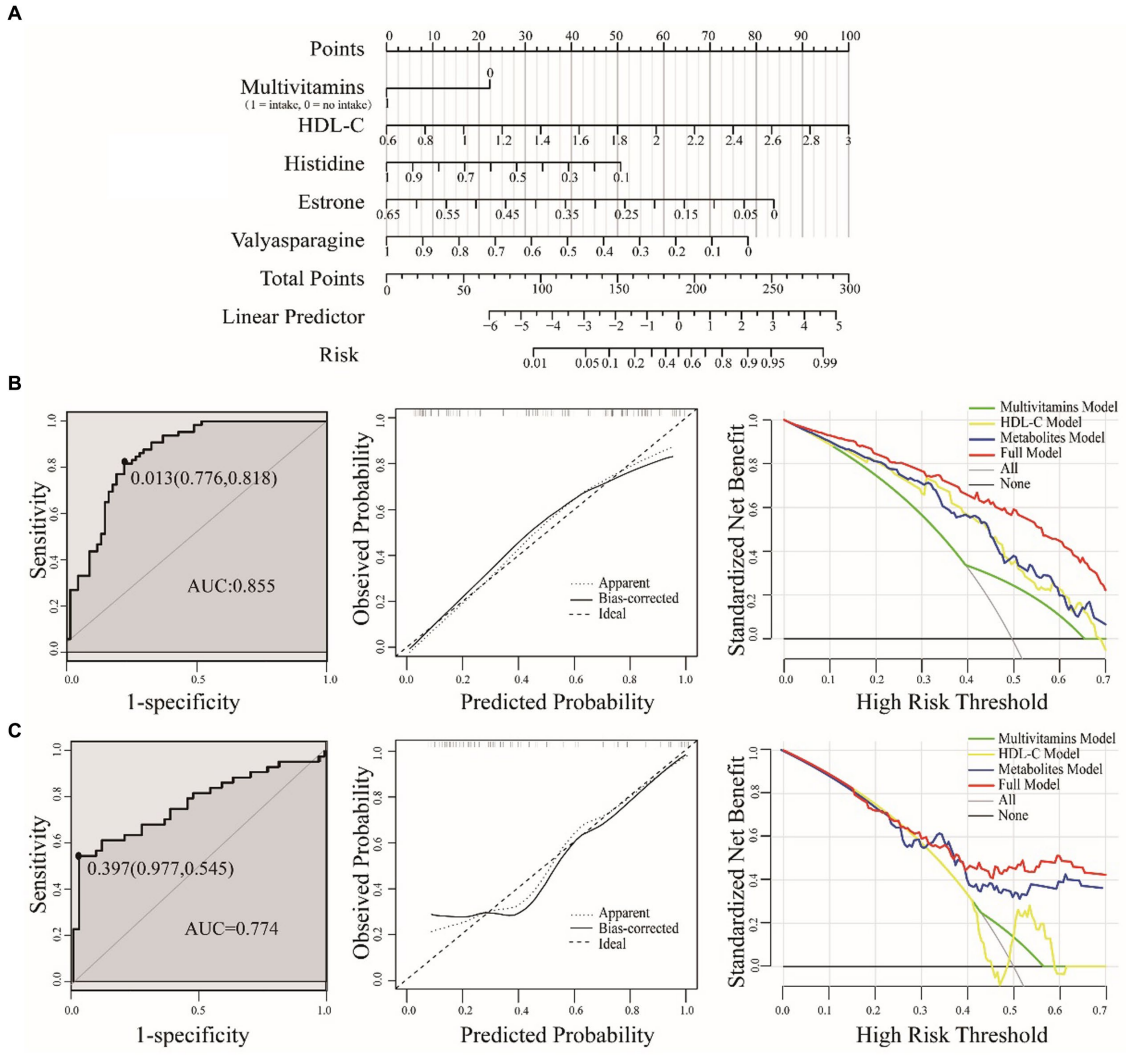
A nomogram for predicting PDS risk **(A)**. The value of each variable was assigned a score, and then the scores for each variable were summed. The sum is located on the total points axis, which allows us to predict the probability of PDS risk. Evaluation of the nomogram model were based on training **(B)** and test sets **(C)**, including ROC, calibration plot and DCA. ROC for the nomogram was generated using bootstrap resampling (1,000 times). The nomogram calibration plot shows the proximity of the solid line (performance nomogram) to the dotted line (ideal model), which serves as an indicator of prediction accuracy. The DCA graph demonstrates the expected net benefit per patient, with the red solid line representing the prediction model, the gray line representing all patients with PDS, and the solid horizontal line indicating the absence of PDS patients.

For the development of optimal nomogram prediction model, we first sought to identify the clinical factors and metabolic biomarkers with the most effective predictive power. Through logistic regression models, we identified five predictors associated with PDS: multivitamin intake, HDL-C, histidine, estrone and valylasparagine. These predictors were selected from multivitamin supplementation, clinical blood indicators and metabolic biomarkers, respectively. We then constructed a risk prediction nomogram (Figure 4A), where the cumulative scores indicated the risk of PDS. The C-index of the nomogram was 0.855, affirming its robust predictive performance.

To validate the accuracy of the nomogram, we employed the test set. Using bootstrap methods with 1,000 resamples, the area under the curve (AUC) for the training and test set nomogram was determined to be 0.855 (95% CI, 0.791–0.920) and 0.774 (95% CI, 0.673–0.874), respectively. We also established the corresponding cut-off values for the training and test sets as 0.013 and 0.397. In practical terms, when the cumulative threshold of the five indicators in the training set reached 0.013, the model demonstrated optimal discriminative power. The same principle applied to the test set. To assess the clinical utility of the model, we employed DCA, which quantifies the net benefit at various threshold probabilities within the dataset. In both the training and test sets, our full model consistently outperformed single-factor models, indicating its great potential for clinical applications (Figure 4).

## Discussion

The screening of PDS is important in the management of perinatal mental health. Up to now, the predictive power of single risk factors is limited. In this study, we investigated the serum metabolome, multivitamin supplementation factors, and clinical blood indicators to develop an integrated predictive model for assessing the risk of PDS. Our approach involved several key steps. Firstly, we used non-targeted metabolomics technique to identify 36 differential metabolites distinguishing PDS from NPDS, and combined multiple models (DSPC network analysis, 12 topological methods from Cytohubba) to rigorously identify 6 key metabolites as metabolic biomarkers. Secondly, we conducted the logistic regression analysis, and identified multivitamin use as a protective factor for depressed pregnant women. Thirdly, we employed MFA models to examine the association of subscales of depressive symptoms with serum metabolic biomarkers, supplementation factors. Finally, we developed and validated a nomogram to assess the risk of PDS. This study offers a promising method for improving the early identification and management of PDS.

### Biological significance of the serum metabolome in PDS

Previous studies have identified abnormal levels of phenylalanine, tyrosine, glutamate, and leucine as diagnostic or predictive factors for depression (13, 14, 20). In this study, we also observed the disruption of amino acid metabolism in PDS group, which was consistent with previous studies. Histidine showed a more noticeable decreasing trend in the PDS group. Histidine is a semi-essential amino acid that needs to be supplemented through diet. It is converted to histamine, a potential new target for depression treatment of (38) by the enzyme decarboxylase. Intriguingly, while histamine barely penetrates the blood-brain barrier, histidine can be transported into the brain via histidine transporter proteins. Hence, brain histamine needs to be synthesized within the central nervous system rather than supplied by peripheral tissues (39) As a precursor of histamine, histidine is critical for maintaining brain histamine levels. Studies have shown that oral histidine oral histidine improves working memory in mice and significantly improves fatigue, sleep quality, mood state scores, decreases reaction time, and increases the perception of clear thinking in human subjects (40).

Lipids also play a crucial role in the pathology of depression (41). We observed that alpha-linolenic acid (ALA), anandamide (arachidonoyl ethanolamide, AEA), estrone and sphingosine were associated with depressive symptoms. ALA, an omega-3 essential fatty acid, can be converted into DHA, AA, EPA, and other important compounds in the body (42). Although the effect of omega-3 on the pathophysiology and treatment of depression remains obscure, cohort studies and meta-analyses have demonstrated that reduced intake of omega-3 food and ALA is associated with an increased risk of depression (43–45). There is no consensus on the changes in AEA in depressed mood. AEA is an important endogenous cannabinoid that exerts its activity by activating cannabinoid CB1 and CB2 receptors (46). Studies have shown that taking cannabinoids can reverse depressive-like behaviors (47). The opposite result has been seen in other experiments: women diagnosed with major depression had reduced AEA concentrations, but women with mild depression had increased AEA concentrations, which the authors hypothesized was the body’s protective buffer against disease progression (48) And AEA it is more widely known for its regulation of obesity and its involvement in energy metabolism in the brain and peripheral tissues. There are many studies showed an increase in serum AEA during intake of high-fat diets and weight gain exactly consistent with the context of pregnancy-related physiological changes (49). Estrone is an important estrogen that affects the vulnerability of specific brain regions, and fluctuating levels (particularly estrogen withdrawal) make women of childbearing age more susceptible to mood disorders (50). During pregnancy, estrogen levels usually increase steadily with each week of pregnancy. Nevertheless, estrone levels were significantly lower in the PDS group, and there may be a process of decreasing hormone levels, suggesting that mood disorders may be related to pregnancy hormones. Lastly, sphingosine, a degradation product of ceramide, can be synthesized into sphingomyelin with lipoyl CoA and phosphorylcholine. Previous research has reported a negative association between sphingomyelins and depression among American Indians with depression (51). Sphingosine can also be phosphorylated to sphingosine-1-phosphate (S1P), which is abundant in brain tissue. Knockdown of S1P-related receptors (S1PR2 and S1PR3) increased both anxiety and depression levels (52), while overexpression of S1PR3 not only attenuated anxiety-related and depression-related behaviors, but also promoted the production of adrenocorticotropic hormone (ACTH) to increase the adaptability of rats to stressors (52, 53).

### Multivitamin supplement associates with PDS

Our study yielded a significant finding—the protective effect of multivitamin use against PDS. To achieve this, we employed multiple factor analysis (MFA), a powerful technique that enables the examination of subjects using multiple sets of variables while considering the interplay among these variables (54). It is the first time such an analysis has been applied in prenatal depression research.

The MFA analysis revealed that histidine, estrone, sphingosine, and multivitamin intake were all negatively associated with three clinical symptoms, with histidine also demonstrating a positive association with multivitamin use. Earlier studies have shown that individuals who took daily mineral and vitamin complex supplements experienced significant improvements in overall cognition and specific functions such as memory and executive function (e.g., planning and decision making) (35). Moreover, the mothers who took several micronutrients had significantly lower EPDS scores during the first and second trimesters (36). These findings suggest the existence of a supplement-metabolite-symptom-relevance chain, wherein exogenous vitamins influence the body’s state of various depressive symptoms by modulating specific metabolites. Consequently, nutritional interventions may serve as an adjunct treatment in PDS, offering potential benefits without reported side effects. It’s important to note that nutritional deficiencies often involve a combination of nutrients rather than a single nutrient due to the diversity of the typical diets.

### Clinical blood indicators associate with PDS

Our study revealed abnormalities in lipid-related indicators: total cholesterol, triglycerides, LDL-C and HDL-C. These findings are partly consistent with some of previous research in the field of depression studies (55, 56). However, the specifical link between blood lipids and pregnant women with depression is unclear. HDL-C may also have negative health effects and show a U-shaped curve with it. Simple measurement of HDL-C concentration does not yield information about HDL function (57) and measurement of “dysfunctional” HDL may be an alternative therapeutic target (58). Therefore, lipid concentrations may be a more distal marker of elevated risk for depression. Further investigation into this relationship could provide valuable insights into the mechanisms underlying prenatal depression and its potential treatment strategies.

### Predication models of PDS risk

In developing a robust risk prediction model, it is essential to ensure that multimodal data provide a few key variables while excluding numerous confounding factors. To achieve this, we utilized multiple models for hierarchical screening, ultimately identifying six key metabolic biomarkers. The nomogram model also integrated protective factors like multivitamin use and conventional blood indicators.

While perfect models are hard to attain, the integration of various models with distinct experimental designs can enhance validity compared to relying on a single model. This approach is not only applicable in the field of depression but is a trend in diagnosis of complex diseases. Researches have consistently shown that multi-model fusion model yields superior results in comparison to single prediction methods, offering improved performance and higher accuracy (59, 60). In this study, we used several models to identify the most representative metabolic biomarkers. The combined use of models can complement each other’s performance, thus reducing the uncertainties and inaccuracies associated with each individual model.

Several biomedical technologies, including omics, have attempted to identify reliable biomarkers of depression to facilitate screening. For example, microRNAs have been detected in body fluids, and magnetic resonance imaging (MRI) has demonstrated sensitivity and specificity greater than 80% (28, 29). Unfortunately, many of these models performed poorly when they were validated in test sets or other populations, perhaps due to the inherent heterogeneity of the disease. Thus, a critical aspect in developing disease risk prediction models is to gather comprehensive multimodal data from patients, allowing for the precise assessment of individual characteristics, the inclusion of more risk factors, and the use of more accurate laboratory testing techniques (15). In this study, our model, encompassing information at three levels—multivitamin supplementation, clinical blood indicators, and metabolome—offers a more precise prediction of the risk of depressive symptoms. The results from the DCA matched expectations, with the full model significantly outperforming a single factor in predicting PDS. One prominent example of a similar approach is the North American prodrome longitudinal study, which collected detailed baseline measures and information across five domains: genomics, hormones, anatomy, physiology, and behavior, finally developed a robust prediction algorithms (61).

Nomograms have been used for the reliable prediction of various aspects of depression risk factors (e.g., demographic characteristics, social factors, and biological factors) (62, 63). However, there are limited reports on the use of nomograms in the PDS, and most reports typically focused on a single domain of risk factors (64). Therefore, we have constructed a risk prediction nomogram model for PDS by combining metabolic biomarkers, dietary factors, and clinical blood indicators. We have evaluated the model’s accuracy in a test set, demonstrating its strong predictive capabilities and clinical applicability. The risk assessment model has great potential, because these lifestyle factors (multivitamin supplement factors), and routine clinical examination indicators are readily available, quantifiable, and easy to interpret.

### Limitations

Our study has some limitations. First, the sample size was relatively small, which limited the precision and statistical power of the study. Therefore, the results should be interpreted with caution. Future research with larger cohorts and possibly multicenter collaborations would not only enhance the statistical power but also increase the diversity of the study population, which can lead to more robust and widely applicable results. Second, the study did not consider various other lifestyle and supplementation factors, thus potential biases might be introduced into the observed results. Third, using a single metabolomics platform limited the scope of the metabolic information obtained. Employing multiple platforms in future studies could capture comprehensive metabolome, leading to a richer and potentially more accurate metabolic profile associated with PDS.

## Conclusion

This study has offered valuable insights into the protective factors and routine clinical indicators associated with PDS. Utilizing non-targeted metabolomics techniques and a multi-model screening approach, we have identified six crucial metabolites differentiating PDS from NPDS. We have further explored the relationships between these characteristic variables to build a robust risk prediction model. These findings significantly enhance our understanding of prenatal depression’s pathogenesis. Distinguishing from previous studies on depression during pregnancy, our research’s comprehensiveness is underlined by the integration of a multi-model approach and the utilization of multimodal data. This approach has enabled precise screening of biomarkers and the development of powerful predictive models. The findings of this study mark an important step forward in prenatal depression research, opening new avenues for future investigation, and have the potential to inform improved screening and intervention strategies.

## Data availability statement

The original contributions presented in the study are included in the article/Supplementary material, further inquiries can be directed to the corresponding author.

## Ethics statement

The studies involving humans and the protocols of this study were approved by the Medical Ethics Committee of the First Affiliated Hospital of Xiamen University (Approved ID: 2021-Research No. 050). The studies were conducted in accordance with the local legislation and institutional requirements. The participants provided their written informed consent to participate in this study.

## Author contributions

RY: investigation, data curation, formal analysis, visualization, software, and writing—original draft. ZL: software, formal analysis, and visualization. YC: investigation, data curation, and validation. NC: investigation, data curation, and methodology. YZ: investigation, supervision, and resources. JZ: conceptualization, supervision, resources, project administration, and writing—review and editing. GH: conceptualization, supervision, resources, project administration, and funding acquisition. All authors contributed to the article and approved the submitted version.

## Footnotes

^1^ http://www.metaboanalyst.ca
