# Supplementary material for: Assessing the risk of prenatal depressive symptoms in Chinese women: an integrated evaluation of serum metabolome, multivitamin supplement intake, and clinical blood indicators
Source: Front Psychiatry. 2024 Jan 11;14:1234461. doi: 10.3389/fpsyt.2023.1234461 (PMC10808622; doi:10.3389/fpsyt.2023.1234461)
Supplement: Supplementary file 1 [file Data_Sheet_1.pdf]

Supplementary materials

Figure S1. PCA score plot of the samples and QCs.
Figure S2. Serum Metabolome Analysis.
Figure S3. Results of multiple factor analysis.
Table S1. Baseline characteristics of training set and test set.
Table S2. Risk factors derived from logistic regression with prenatal depression symptoms as the dependent variable.

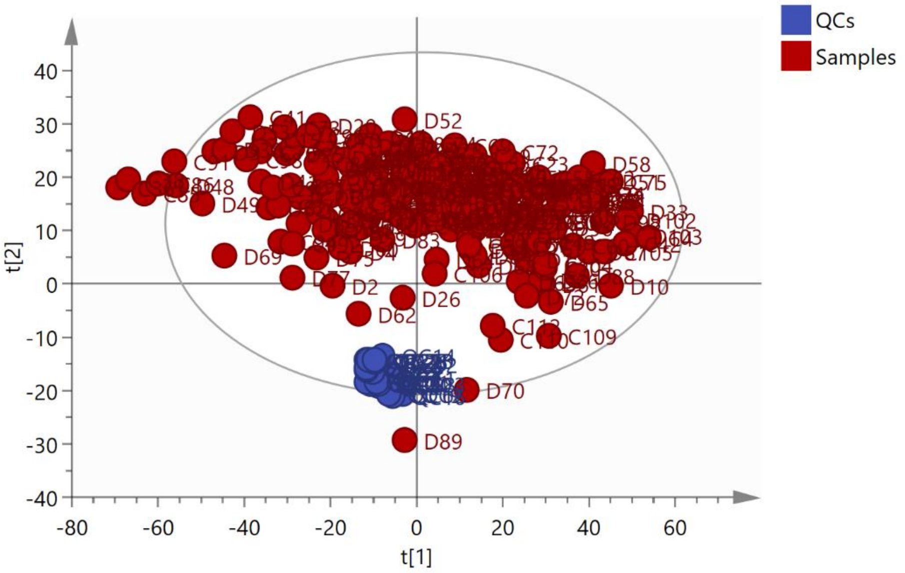

FIGURE S1 | PCA score plot of the samples and QCs.

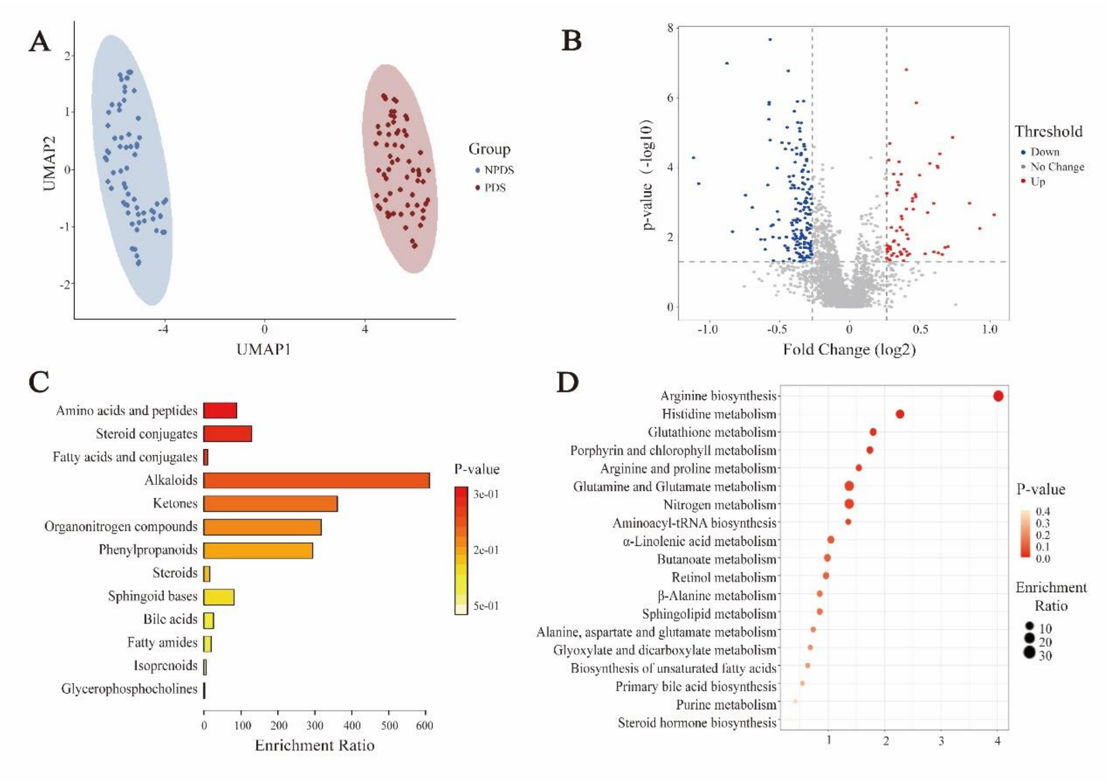

FIGURE S2: Serum Metabolome Analysis. **(A)** UMAP plot of the serum metabolome of the participants. **(B)** Volcano plot depicting differential metabolites between the PDS and NPDS groups. Non-significant metabolites are indicated in gray, while significantly up-regulated and down-regulated metabolites are shown in red and blue, respectively. **(C)** Bar chart displaying the main-class chemical structures of each metabolite set. **(D)** Dot plot providing an overview of the metabolic pathways enriched by the KEGG library.

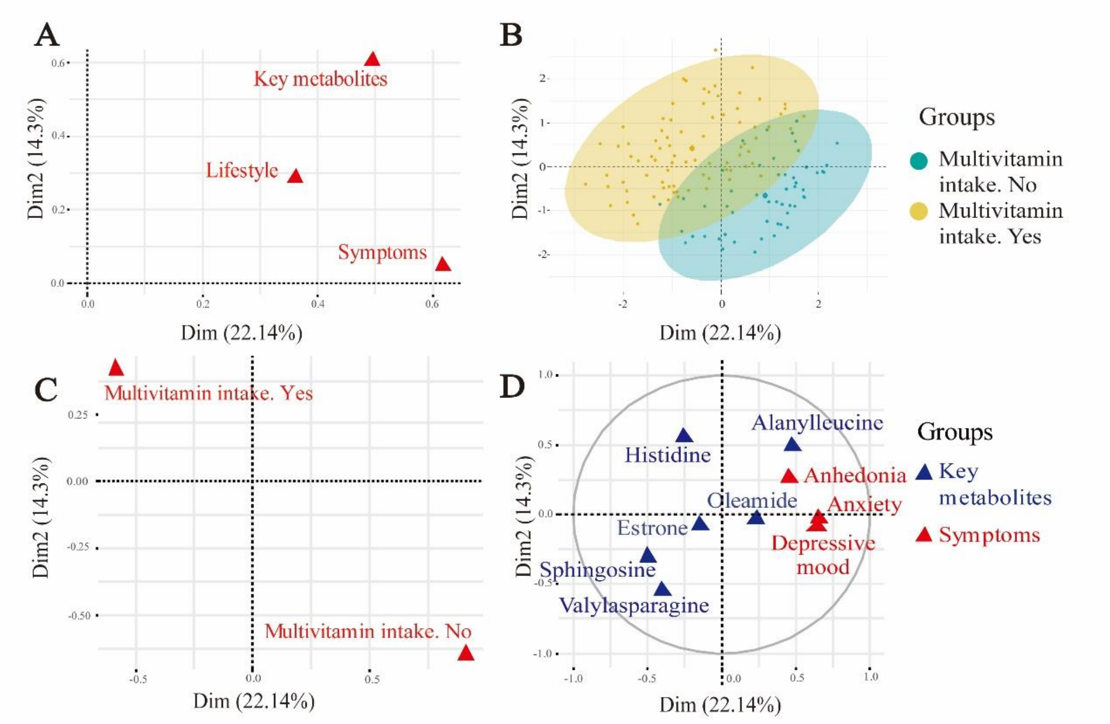

FIGURE S3 | Multiple factor analysis. Dim1 and Dim2 indicate the contributions of variables on two dimensions.

The variable set as a whole was calculated for its correlation with each MFA axis, with ranked coordinates reflecting the degree of correlation of the variable sets with each MFA axis **(A)**. Scatterplot of the two groups with and without multivitamin intake **(B)**. Figures C and D show the distribution of quantitative and qualitative variables on the axes **(C and D)**. The quantitative variables are shown as vectors and each triangle represents the relative position of the corresponding quantitative variable in circle. The variable colors in the figure represent the variable sets to which the variables belong. If the angles between two variables are acute, they are positively correlated; if they are obtuse, they are negatively correlated; if they tend to be orthogonal, there is no correlation. The smaller the angle between a variable and an axis, the greater its contribution to that axis; the longer the length of the projection of a variable on an axis, the greater its contribution to that axis.

Table S1 | Baseline characteristics of training set and test set

Characteristics	Training set			Test set		
	NPDS (n = 67)	PDS (n = 66)	*p*	NPDS (n = 44)	PDS (n = 44)	*p*
Age,[a] median (IQR)	31.0 (29.0–35.0)	31.0 (28.0–35.0)	0.894	31.0 (28.0–33.0)	30.0 (27.0–33.5)	0.391
Prepregnancy BMI,[a] median (IQR)	20.4 (19.0–22.3)	20.5 (18.7–22.6)	0.970	20.4 (18.0–23.0)	21.5 (18.9–22.8)	0.230
Pregnancy BMI,[a] median (IQR)	25.2 (23.4–27.3)	25.2 (23.6–27.0)	0.717	25.9 (23.4–26.9)	26.5 (24.1–28.8)	0.367
Gestational weeks, [a] median (IQR)	35.0 (33.0–37.0)	34.0 (33.0–36.0)	0.171	35.0 (33.0–36.0)	35.0 (33.0–37.0)	0.973
Birth parity,[b] n (%)			0.438			0.375
No	38 (56.7)	33 (50.0)		14 (31.8)	18 (40.9)	
Yes	29 (43.3)	33 (50.0)		30 (68.2)	26 (59.1)	
EPDS score,[a] median (IQR)	6.0 (4.0–7.0)	11.0 (11.0–12.0)	<0.001	6.0 (4.0–7.0)	11.0 (11.0–12.0)	<0.001
Anhedonia,[a] median (IQR)	2.0 (1.0–3.0)	3.0 (2.0–4.0)	<0.001	2.0 (1.0–3.0)	3.0 (2.0–3.0)	<0.001
Anxiety,[a] median (IQR)	3.0 (2.0–4.0)	6.0 (5.0–6.0)	<0.001	3.0 (2.0–4.0)	5.5 (4.5–6.0)	<0.001
Depressive Mood,[a] median (IQR)	1.0 (0.0–2.0)	3.0 (2.0–4.0)	<0.001	1.0 (0.0–2.0)	3.0 (3.0–4.0)	<0.001

Data presented as median (IQR), or n (%). IQR interquartile range

[a]Analyzed by the Mann–Whitney U test.

[b]Analyzed by Chi-square test.

Table S2 | Risk factors derived from logistic regression with prenatal depression symptoms as the dependent variable.

Factors	Single-factor logistic regression		Multi-factor logistic regression	
	p	OR（95% *CI*）	*p*	OR（95% *CI*）
Age	0.918	0.996（0.921-1.077）		
Pregnancy BMI	0.812	0.988（0.897-1.089）		
Pre-pregnancy BMI	0.710	1.018（0.928-1.116）		
Gestational week	0.223	0.918（0.801-1.053）		
Birth parity	0.438	1.310（0.662-2.594）		
Passing smoking	0.491	1.348（0.577-3.152）		
Drinking	0.988	1.016（0.139-7.431）		
Chemical material exposure	0.529	1.471（0.442-4.893）		
Multivitamins supplementation	0.004	0.346（0.168-0.713）	0.005	0.326（0.149-0.713）

DHA supplementation	0.136	0.592（0.297-1.180）
Iron supplementation	0.229	0.627（0.293-1.341）
Zinc supplementation	0.327	3.143（0.318-31.015）
Calcium supplementation	0.603	0.776（0.299-2.016）
Deepsea fish supplementation	0.791	1.038（0.786-1.372）
Vegetable oil supplementation	0.867	0.955（0.559-1.631）
